# Mapping the in situ microspatial distribution of ice algal biomass through hyperspectral imaging of sea-ice cores

**DOI:** 10.1038/s41598-020-79084-6

**Published:** 2020-12-14

**Authors:** Emiliano Cimoli, Vanessa Lucieer, Klaus M. Meiners, Arjun Chennu, Katerina Castrisios, Ken G. Ryan, Lars Chresten Lund-Hansen, Andrew Martin, Fraser Kennedy, Arko Lucieer

**Affiliations:** 1 Institute for Marine and Antarctic Studies, College of Sciences and Engineering, University of Tasmania, Private Bag 129, Hobart, TAS 7001 Australia; 2 Australian Antarctic Division, Department of Agriculture, Water and the Environment, Kingston, TAS 7050 Australia; 3 Australian Antarctic Program Partnership, Institute for Marine and Antarctic Studies, University of Tasmania, Hobart, TAS 7001 Australia; 4 Max Planck Institute for Marine Microbiology, Celsiusstr. 1, 28359 Bremen, Germany; 5 Leibinz Center for Marine Tropical Research, Fahrenheitstrasse 6, 28359 Bremen, Germany; 6 School of Biological Sciences, Victoria University of Wellington, PO Box 600, Wellington, New Zealand; 7 Aquatic Biology, Department of Bioscience, Aarhus University, Ole Worms Allé 1, Building 1134, 8000 Aarhus C, Denmark; 8 Arctic Research Centre, Aarhus University, Ny Munkegade 116, Building 1540, 8000 Aarhus C, Denmark; 9 Discipline of Geography and Spatial Sciences, School of Technology, Environments and Design, College of Sciences and Engineering, University of Tasmania, Private Bag 76, Hobart, TAS 7001 Australia

**Keywords:** Microbial ecology, Marine biology, Optical imaging, Imaging and sensing, Optical spectroscopy, Biofilms, Data acquisition, Image processing, Statistical methods

## Abstract

Ice-associated microalgae make a significant seasonal contribution to primary production and biogeochemical cycling in polar regions. However, the distribution of algal cells is driven by strong physicochemical gradients which lead to a degree of microspatial variability in the microbial biomass that is significant, but difficult to quantify. We address this methodological gap by employing a field-deployable hyperspectral scanning and photogrammetric approach to study sea-ice cores. The optical set-up facilitated unsupervised mapping of the vertical and horizontal distribution of phototrophic biomass in sea-ice cores at mm-scale resolution (using chlorophyll *a* [Chl *a*] as proxy), and enabled the development of novel spectral indices to be tested against extracted Chl *a* (R^2^ ≤ 0.84). The modelled bio-optical relationships were applied to hyperspectral imagery captured both in situ (using an under-ice sliding platform) and ex situ (on the extracted cores) to quantitatively map Chl *a* in mg m^−2^ at high-resolution (≤ 2.4 mm). The optical quantification of Chl *a* on a per-pixel basis represents a step-change in characterising microspatial variation in the distribution of ice-associated algae. This study highlights the need to increase the resolution at which we monitor under-ice biophysical systems, and the emerging capability of hyperspectral imaging technologies to deliver on this research goal.

## Introduction

Sea ice is a porous multiphase medium whose interstitial environment is inhabited by diverse phototrophic and heterotrophic microbial communities^1^. Phototrophic ice algae dominate ice-associated biomass and contribute significantly to the overall primary production of ice-covered waters and serve as critical food source for marine herbivores^2–5^. The dynamic and multiphase nature of sea ice imposes strong horizontal and vertical gradients in temperature, salinity, porosity and light transmittance^6,7^, all of which influence the in situ distribution of algal cells^2,8^. Biological properties of ice algal communities such as abundance, species composition and photosynthetic rates are thus extremely variable over time and at spatial scales from millimeters to kilometers^8–10^. While most of the algal biomass is concentrated at the ice-water interface, microbes are also present in the interior of the ice matrix, which adds to the biocomplexity of the system^2,8^. Measurement capability to determine the mm-scale spatio-temporal distribution of algal biomass in sea ice is currently lacking, thereby limiting a mechanistic understanding of its environmental drivers which is needed to inform predictive modelling and strategic sampling.

In particular there is the lack of efficient methods capable of non-invasively tracking algal biomass across different scales, both vertically and horizontally, and concurrently with its physical drivers^11,12^. There is now evidence that variation in under-ice biophysical properties can range from the microscale (0.001 m^2^) to the mesoscale (10 m^2^)^13–15^, and this cannot be satisfactorily resolved using point-based sampling methods^16,17^. Traditional and emerging sea-ice field sampling methods include ice coring^18^, under-ice bio-optical sensing techniques via L-shaped deployment arms^19–21^ or unmanned underwater vehicles (UUVs)^9,10,22,23^. The use of under-ice optical sensing from UUVs has extended the spatial coverage of algal surveys (e.g., covering meso- to floe-scale areas), but resolutions still remain coarse as a result of large footprints of underwater radiance (or irradiance) sensors^10,16,22^. This shortcoming demonstrates a gap in field-sampling techniques that permit the quantification of horizontal and vertical distributions and temporal dynamics of ice algae biomass at relevant spatial scales.

Underwater Hyperspectral Imaging (HI) is one method that could deliver a methodological turning-point for quantitative mapping of fine-scale sea-ice biophysical conditions that is relevant to larger scale analyses^24,25^. HI can be used to quantify biogeochemical properties of a target in each spectrally-resolved pixel within an image^26–28^. In situ HI has revolutionized the scales of observation on both terrestrial^29–31^ and marine^32–34^ ecosystems. HI of extracted samples (ex situ) can be used to better understand the target spectral behaviour and to create a baseline measurement with the data acquired in situ. This enables detailed understanding of a particular target’s interaction with light but also allows us to capture dimensions and dynamics that are not visible from the in situ surface perspective (e.g., vertical variability). Some recent examples of ex situ applications include scanning of glacial ice cores to detect chemical impurities^35^, scanning of soil cores to map fine-scale organic carbon hotspots^36^, and scanning of sediments to determine pigment concentrations in microbial phototrophs^37,38^.

We have recently demonstrated how HI can qualitatively capture biomass variability at the sub-mm spatial resolution in both artificial laboratory ice^24^ and in situ under Antarctic fast ice^25^. In this study, we advance the use of HI technology to map the fine-scale vertical and horizontal distribution of sea-ice algae through optical quantification of the photosynthetic pigment chlorophyll *a* (Chl *a*). Traditional and novel spectral indices, established from transmittance measurements of ice-core sections, were developed and correlated with fluorometrically derived Chl *a* values from the ice cores. The retrieved bio-optical regression models were then applied to hyperspectral imagery acquired both in situ, using an under-ice HI scanning platform, and ex situ to the extracted ice cores. The resulting maps provide the first spatially explicit quantitative estimates of Chl *a* concentration, illustrating that HI is a critical developmental step in our capacity to make scalable under-ice observations for sea-ice algae.

## Materials and methods

### Study area and ice coring

A field camp was established at Cape Evans, Antarctica (77.637° S, 166.401° E), from the 14th of November to the 5th of December 2018. The sea ice across the study area had a thickness of 180 ± 1 cm, except for occasional ridged or cracked areas. The distinct under-ice biophysical environment was visually explored using a Seabotix LBV-300 remotely operated vehicle (ROV) (Teledyne Marine, Seabotix, California, USA). Sea-ice surface conditions in the area were typically snow-free apart from patches of 0.5–1 cm hard old snow layers or 1 to 5 cm compacted snow reliefs throughout the 21-day study period.

Forty-two ice cores were extracted using a Kovacs Mark V ice corer (14 cm internal diameter) between the 19th of November to the 2nd of December. Of these, 22 were taken from bare ice (snow free) areas, 12 from the area with 0.5–1 cm thin snow cover, 7 cores on the 1–5 cm snow drifts, and 1 core from an area with a 10 cm snow patch. After retrieval, the bottom (~ 60 cm) of each core was immediately cut off and placed into a black plastic bag to protect it from sunlight and promptly taken into a darkened field laboratory for image acquisition and biological processing.

**Figure 1:**
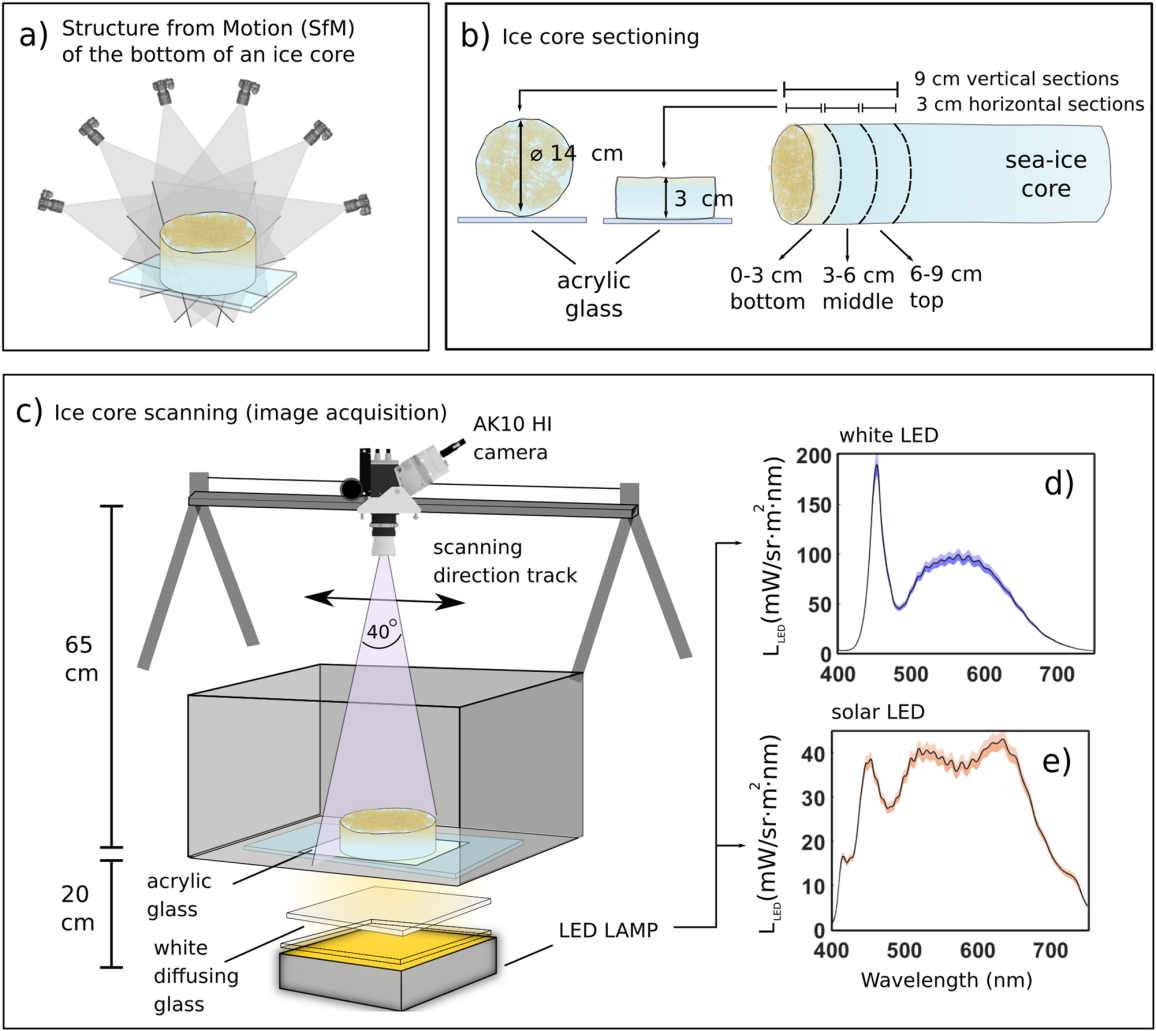
An overview of sample processing and the hyperspectral imaging optical set-up. Specifications and technical details are found in the supplementary material. (**a**) 3D model reconstruction using structure from motion (SfM) digital photogrammetry on horizontal bottom cores sections to retrieve microtopography. (**b**) Vertical and horizontal core samples preparation for hyperspectral image acquisition. A total of 6 vertical scans (of 9 cm length) and 54 horizontal scans (3 cm thick) were acquired in this study. Samples are imaged within a dark box. (**c**) The ice core scanning set-up based on transmitted and artificial homogenously diffuse illumination. AK10 refers to the pushbroom camera model Aisa Kestrel 10 from Specim. (**d**) and (**e**) illustrate respectively the mean ± standard deviation of radiance (L) emitted by the white and solar LED lamps utilised for illuminating the samples. The LEDs were set to emit an E_d, PAR_ of < 30 µmol photons m^−2^ s^−1^ to avoid potentially photo-damaging the algal communities, which are typically low-light adapted.

The ice water interface micro-topography of our study site was revealed using structure from motion (SfM) digital photogrammetry on selected ice core bottoms (0.015 m^2^ area) (Fig. 1a). SfM provides highly resolved and scaled 3D models of objects or surfaces using a set-of overlapping pictures and photogrammetric software^39,40^. Imagery of the bottom surfaces was collected with a Nikon D500 digital camera and Tamron SP 90 mm F/2.8 Di MACRO 1:1 VC USD macro lens from different angles and perspectives. We used Agisoft Metashape software for processing and followed standard workflows as outlined in the software manual^41^. Known core lengths were used to scale the models.

### Hyperspectral image acquisition

Sea ice is a translucent medium that allows the retrieval of some of its bio-optical properties from measurements in transmittance mode^42,43^. We devised an optical configuration to measure transmitted radiation along the vertical axis of the bottom 9 cm, and horizontally on 3 cm thick sections, of the ice cores at high spectral (1.7 nm) and spatial (≤ 0.07 cm) resolution (Fig. 1b,c). The system further utilized the horizontal section scans to develop bio-optical predictive models relating spectra to extracted ice algal biomass. Sensing of transmitted, rather than reflected radiance, emulates under-ice close-range bio-optical remote sensing approaches to retrieve estimates of Chl *a* in sea ice^9,20^. Specifications and technical considerations of the scanning approach are found in the supplementary material. In situ hyperspectral images were captured beneath the sea ice area from which the ice cores were sampled. We used an underwater HI and photogrammetric payload mounted on an under-ice sled fully described in a previous study^25^.

#### Vertical ice-core sections

Vertical scans were prepared for the bottom 9 cm of 6 of the 42 ice cores (Fig. 1b). Hyperspectral imaging frequency was set to 10 Hz with an integration time ranging from 90 to 99 ms with a sliding rail speed of ∼0.4–0.5 cm s^−1^. We did not apply any in-camera spectral binning and this resulted in a native spectral sampling interval of 1.7 nm. Spatial binning was applied to reduce the image from 2048 to 1024 pixels across the scanning direction (Fig. 1) to boost signal to noise ratio (SNR). The entrance pupil of the camera was located approximately 55 cm from the centre line of the core (Fig. 1c). Across-track scan lines were ~ 40 cm with a spatial resolution of 0.39 mm and vertical cores width covered 360 pixels over the across-track scan line. The samples were illuminated with a solar simulating spectrum LED light (Fig. 1e).

#### Horizontal ice-core sections

Horizontal ice-core sections were prepared by cutting off the lower-most 3 cm section from the core (Fig. 1b). This sampling was done for all 42 cores; thus, all bottom 0–3 cm sections were imaged. Six selected cores were processed by sectioning the core at 3 cm intervals starting at the ice water interface at 0–3 cm, 3–6 cm and 6–9 cm (Fig. 1b). This procedure provided us with an additional twelve horizontal core sections for scanning, yielding a total of 54 horizontal core samples and allowing us to explore the horizontal variation of biomass deeper into the ice column, and increasing our samples size and biomass range.

The sections were placed with the ice-water interface facing towards the scanner (Fig. 1c). No in-camera spectral binning was applied, yielding a native spectral sampling interval of 1.7 nm, and spatial binning was applied as per vertical scans. Two different LEDs (Fig. 1d,e) were used for imaging, and image acquisition parameters were adjusted as per different light intensities (see supplementary material for further information). Eighteen sections were scanned using the white LED. Imaging frequency was set to 15–20 Hz with 60–75 ms integration time and a sliding rail speed of ∼0.8–1.1 cm s^−1^. Thirty-six section scans were taken using the solar LED. Imaging frequency was set to 10 Hz with integration times ranging from 90 to 99 ms and a sliding rail speed of ∼0.4–0.5 cm s^−1^. The distance between the camera lens and the core surface was ∼62 cm achieving an across-track scan line of ∼ 45.6 cm and a spatial resolution of 0.44 mm which resulted in around 80,500 pixels per horizontal core surface area of 0.015 m^2^.

#### Under-ice in situ imaging

We used a tethered under-ice hyperspectral and RGB imaging system to capture 10–30 m transects with the same HI camera as the ice-core scanning set-up^25^. Spatial resolution and spectral dimensions were binned at-sensor yielding a native spatial resolution of 0.624 mm, and a spectral sampling interval of 3.5 nm. For this study, we selected a small 85 × 70 cm image region, namely block B, from one of the sampling transects to exemplify the methodology potential. The selected image region exhibited interesting visual features such as the cavities, algal clump patterns, and reliefs in the under-ice topography.

### Image pre-processing and unsupervised exploration

**Figure 2:**
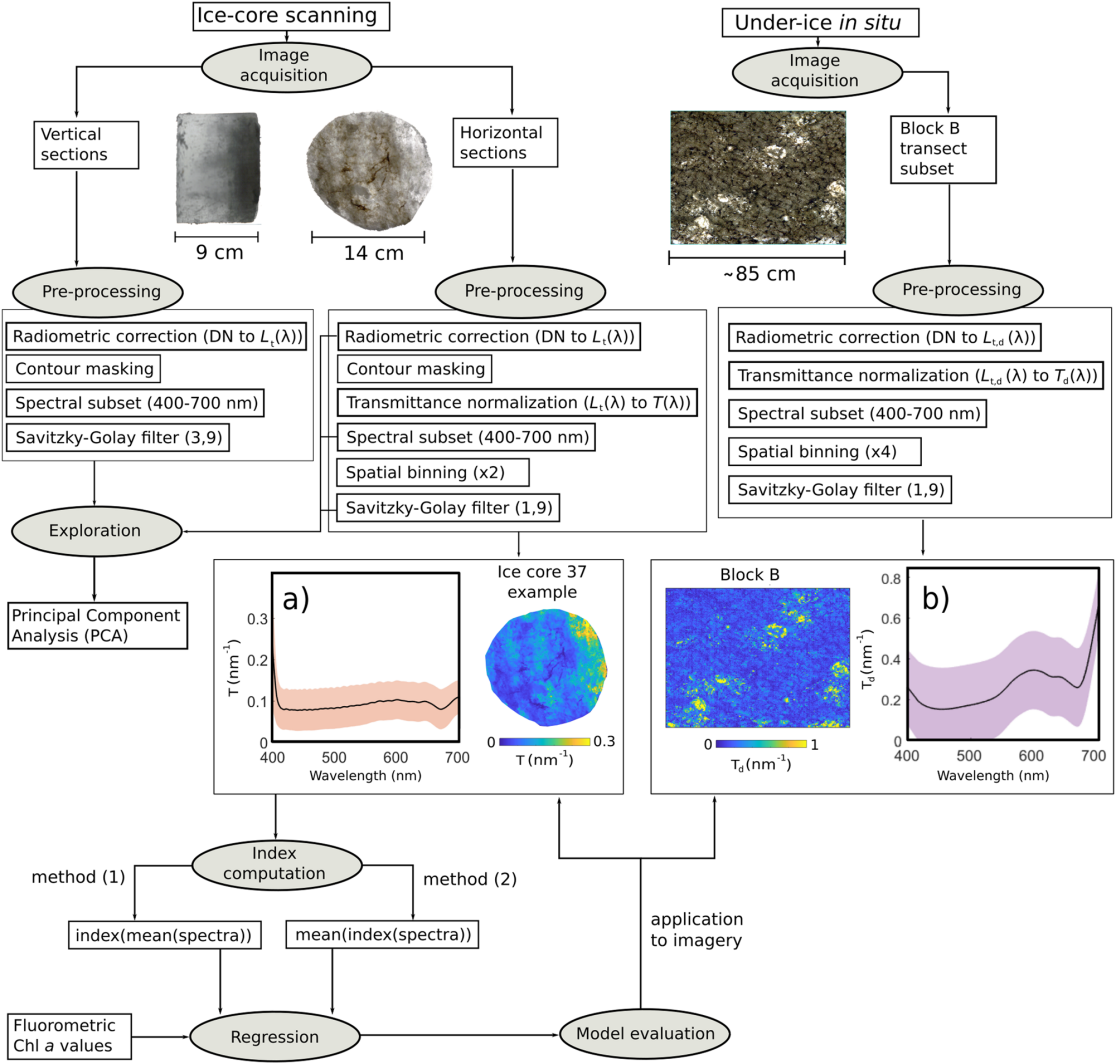
A flowchart of the data pre-processing workflow to yield pseudo-transmittance images and per-pixel biomass estimates from hyperspectral imagery of core sections and in situ. (**a**) and (**b**) display the mean ± standard deviation of directional transmittance at 668 nm through an example ice core (ice core 37) and the under-ice imagery, respectively. The under-ice HI procedure was detailed in a previous study^25^. L_t_(λ) refers to transmitted spectral radiance and T(λ) and T_d_(λ) to spectral transmittance and downwelling spectral transmittance, respectively. DN refers to Digital Number of raw imagery data. Savitzky–Golay filter numbers in parentheses refer to polynomial order and window length in bands, respectively. Two approaches are available to calculate a spectral index for each Sect. (1) from the mean transmittance spectrum of all pixels of the scanned core or (2) from each of the pixels spectrums in the pre-processed pixel of the core and then calculate the mean. Here method (2) was selected. Further information on method (1) can be found in supplementary material.

The image pre-processing and exploration workflow is illustrated in Fig. 2. All of the acquired raw imagery was converted from digital numbers (DN) to radiometric values of transmitted radiance $L_{t}$ (λ, mW m^−2^ sr^−1^ nm^−1^) following standard radiometric correction procedures^25,26^. All horizontal and vertical imagery of cores were manually masked to ensure that only pixels within the ice-core surface were analysed. Spectral sub-setting was applied to keep only photosynthetically active radiation (PAR) (between 400 and 700 nm), which resulted in 179 spectral bands for the core sections imagery and 89 bands for the in situ imagery. This allowed us to focus on Chl *a* absorption features, which improved processing time and reduced noise interference outside of this range.

Principal component analysis (PCA) has been used in HI studies of sea ice to capture per-pixel fine-scale spatial variability of the first two principal components (PCs) scores embodying light intensity variability and biomass proxies in both laboratory artificial sea ice^24^ and in situ^25^. Mean-centred PCA was here employed on the pre-processed imagery of both vertical and horizontal ice-core sections (Fig. 2). Each pixel in the image is represented as a sample in the PCA transformation. For PC scores to be comparable among different images of different cores, all vertical cores were pooled into a common PCA pixel sample pool to derive PC loading factors for the global set of pixels. Analogously, all horizontal core sections were pooled together into a separate pixel sample pool. No PCA was applied to the in-situ imagery of block B as this was explored previously^25^.

Transmitted radiance, $L_{t} \left( \lambda \right),$ of each pixel of the horizontal core sections, was normalized to transmittance by the corresponding averaged LEDs radiance $L_{ LED} \left( \lambda \right)$ as shown in Fig. 1d and e using the following formula:

1 $$T\left( \lambda \right) = \frac{{L_{t} \left( \lambda \right) }}{{L_{ LED} \left( \lambda \right)}}$$

This provides directional transmittance spectra, T(λ), in each pixel of the core surface area (Fig. 2a).

For the image of the in situ block B area, the downwelling radiance spectrum $L_{d,t} \left( \lambda \right)$ was normalized by the average spectrum of specific regions of relatively algae-free cavities $L_{d, cavity} \left( \lambda \right)$ to derive a directional pseudo-transmittance image:

2 $$T_{d} \left( \lambda \right) = \frac{{L_{d,t} \left( \lambda \right) }}{{L_{d, cavity} \left( \lambda \right)}}$$

These algal-free cavity areas were present within the same scene and provide a proxy of light transmittance over roughly the last 1–10 cm of ice bottom (seen in the Fig. 2 in situ image as bright white spots). We aimed to select a cavity that was ≤ 3 cm deep with the minimum possible amount of algae to compare as similarly as possible to the ice-core scanning set-up. To reduce processing times and increase SNR, the block B image was binned spatially in a 4 × 4 array resulting in a resolution of 2.4 mm pixels. Per-pixel smoothing of all the core spectra was carried out using a Savitzky–Golay low-pass filter^44,45^ with a polynomial order of 1–3 (depending if it was a vertical or horizontal section), and window length of 9 bands (Fig. 2). The aim was to reduce noise in the transmitted signals without impacting spectral shapes associated with Chl *a* absorption maximum. The same filtering operation was applied per-pixel to the block B in situ image.

### Pigment quantification and ice algae community assessment

After hyperspectral scanning of the ice-core sections, the samples were left to melt in the dark at 4 °C. After complete melting, the final melt volume was gently homogenized, and 50 mL sub-samples were filtered onto Whatman GF/F filters. The filters were then placed in ethanol for 24 h extraction of Chl *a*. The extracted Chl *a* was measured using a Turner Designs 10AU fluorometer according to standard protocols^18,46^. Volumetric fluorometric Chl *a* estimates (ug L^−1^) were converted to areal concentrations (mg m^−2^) utilizing surface area of the 14 cm diameter core (0.015 m^2^). Sea-ice algae species composition was determined by standard light microscopy (400 × magnification) of melted ice-core samples.

### Spectral estimation of biomass

Spectral indices were computed for each horizontal core section encompassing proxies for phototrophic sea-ice biomass, and the indices were regressed against fluorometrically determined Chl *a* values. The aim was to retrieve regression models that could be applied on a per pixel basis on the pre-processed pseudo-transmittance imagery. In order to retrieve an index value for each core section, two approaches were available: calculate the spectral index (1) from the mean transmittance spectrum of all pixels of the scanned core or (2) from each pixel spectrum in the pre-processed imagery of the core and then calculate the mean (Fig. 2). We selected method (2) for the rest of the analyses as computing indexes based on individual “noisier” pixels from an ice core section will be more representative of the data being acquired in situ. Performances of regression models using method (1) and further information can be found in the supplementary material. The mean directional transmittance spectrum ± standard deviation (sd) of an example horizontal core section (core 37) is shown in Fig. 2a. For comparison, the mean spectrum ± sd of all pre-processed pixels within block B is shown in Fig. 2b.

Sea-ice bio-optical studies have mostly relied on Normalized Difference Indices (NDIs) to relate under-ice transmitted spectra to Chl *a*^21,22^. Here we calculate an NDI for each horizontal ice core section using the following equation:

3 $$NDI\left( {\lambda_{1} ,\lambda_{2} } \right) = \frac{{T_{u} \left( {\lambda_{1} } \right) - T_{u} \left( {\lambda_{2} } \right)}}{{T_{u} \left( {\lambda_{1} } \right) + T_{u} \left( {\lambda_{2} } \right)}}$$

where T_u_ (λ_1–2_) is transmittance at two selected wavelengths λ_1_ and λ_2_. Optimal NDI wavelength were selected by calculating NDIs for all possible wavelength combinations, correlating them with Chl *a* values and plotting them onto a Pearson correlation surface^20^. The two best NDI wavelength combinations were selected based on the following criteria: a good Pearson correlation coefficient (*p* > 0.7 or *p* <  − 0.7), a minimal separation of wavelengths to avoid autocorrelation (> 12 nm ), and different spectral regions that include at least a band from the Chl *a* absorption maxima (e.g., 430–460 nm or 650–700 nm).

Three additional spectral indices targeted at our study area and sensor set-up were designed and tested. Area under the curve (AUC_650–700_)^47,48^, the area under the curve normalised to constant band depth (ANCB_650–700_) and area under the curve normalised to maximum band depth (ANMB_650–700_) which were all calculated from the continuum-removed transmittance spectrum between 650 and 700 nm^47,49^. Continuum removal transformation on the spectrum was used to enhance and standardize the specific absorption features of biochemical constituents^50^. The range 650–700 nm was chosen to include the most sensitive area to the secondary in vivo Chl *a* absorption maximum as seen from the transmittance plots (Fig. 2a,b).

Following continuum removal, we calculated the AUC_650–700_ as:

4 $$AUC_{650 - 700} = \frac{1}{2}\mathop \sum \limits_{j = 1}^{n - 1} \left( {\lambda_{j + 1} - \lambda_{j} } \right)\left( {\rho_{j + 1} + \rho_{j} } \right)$$

where $\rho_{j}$ and $\rho_{j + 1}$ are values of the continuum-removed transmittance at the j and j + 1 bands, $\lambda_{j}$ and $\lambda_{j + 1}$ are wavelengths of the j and j + 1 bands, and n is the number of the used spectral bands. We calculated the ANCB_650-700_ and ANMB_650-700_ index as:

5 $$ANCB_{650 - 700} = \frac{{AUC_{650 - 700} }}{{CBD_{677} }}$$

6 $$ANMB_{650 - 700} = \frac{{AUC_{650 - 700} }}{MBD}$$

where CBD_677_ is a constant band depth of the continuum-removed transmittance, generally at one of the spectrally stable wavelengths of strong chlorophyll absorption, with 677 nm selected in this case. And MBD, is the maximal band depth of the continuum-removed transmittance localized individually for each spectrum.

We also considered the incorporation of a log-transformed index into our index selection as a way to account for exponential attenuation of light intensity being transmitted through a scattering and absorbing medium such as sea ice^33,51^. Thus, we constructed a novel index based on the logarithm of the continuum-removed AUC_650-700_, named LAUC, taking the following form:

7 $$LAUC_{650 - 700} = \log \left( {AUC_{650 - 700} } \right)$$

### Regression models for Chl a

Linear regression analysis was employed to derive bio-optical relationships between integrated Chl *a* measured from spectral indices and extracted Chl *a* data from the horizontal core sections. Natural logarithm transformation was applied to Chl *a* (log(Chl *a* [mg m^−2^])) to deal with the high range of values measured and with the high variance at high Chl *a* values (heteroscedasticity). This transformation is a common approach in sea-ice bio-optical model development and allows for direct comparison across different studies developing indices for under-ice biomass mapping^21,22,48^. The log-linear regression model takes then the following form:

8 $$\log \left( {Chl a} \right) = \alpha + \beta \left( {INDEX} \right)$$

The log-linear regressions were performed for each of the spectral indices and the regressions evaluated through root mean square error (RMSE) and the coefficient of determination (R^2^) for each model. To account for underestimation of the prediction power of the model by the calibration (or training) error, we include adjusted criteria such as the adjusted R^2^ and the Akaike Information Criterion (AIC)^52,53^. For unbiased and reliable model estimation, we performed a tenfold cross-validation (CV)^22,52^, for which the data were subset into 10 different random folds. The fitting of the model and the error calculations were then repeated 10 times, one for each subset. Each time, nine folds (or subsets) of the data were combined to produce a regression model, and then tested to the 10th holdout data fold.

### Quantitative mapping of ice algal biomass and spatial analyses

Based on the results of the statistical analyses, we selected the best performing model and applied it on a per pixel basis to a set of selected transmittance images of horizontal core sections (Fig. 2). The regression models were not applied to the vertical core sections as we considered the optical-geometrical configuration to be too different to the horizontal core sections. However, the best spectral index model was applied to the in situ pseudo-transmittance images from block B to derive a large scale and detailed quantitative Chl *a* abundance map (as per workflow in Fig. 2). We then analysed the in situ biomass map for spatial autocorrelation and complexity to evaluate the captured microspatial variability in a natural ice-algal setting^54^. An empirical variogram was computed to describe the distribution of the Chl *a* abundance with spatial lags of 1.2 cm (5 pixels) and by randomly drawing 10,000 (out of ∼ 76,000) points (pixels) from the block B map. Microscale variability was highlighted by computing the gradient magnitude for every pixel in the image through a Prewitt filter operator. This edge detection filter calculates the maximum rate of change for each pixel in the image in relation to its surrounding pixels.

## Results and discussion

### Chl a biomass and structure of the under-ice habitat at Cape Evans

The broad-scale icescape of Cape Evans portrays the sea ice sub-surface as relatively flat and absent of platelet ice, contrary to what has been experienced in recent studies in nearby locations^16,21,55^. Overall, for the 42 horizontal bottom core Sects. (0– to 3 cm), we observed a mean Chl *a* of 18.74 ± 18.04 mg m^-2^ (range 1.1–117.5 mg m^−2^), in accordance with maximum algal biomass ranges reported for bottom ice in McMurdo Sound^2^. The six horizontal mid-core Sects. (3– to 6 cm) yielded a mean Chl *a* of 0.61 ± 0.4 mg m^−2^ (range 0.13–1.2 mg m^−2^). Finally, for the six horizontal top core sections (6–9 cm), the mean Chl *a* was 0.64 ± 0.48 mg m^−2^ (range 0.14–1.35 mg m^−2^) (see supplementary Fig. 1 for histograms).

**Figure 3:**
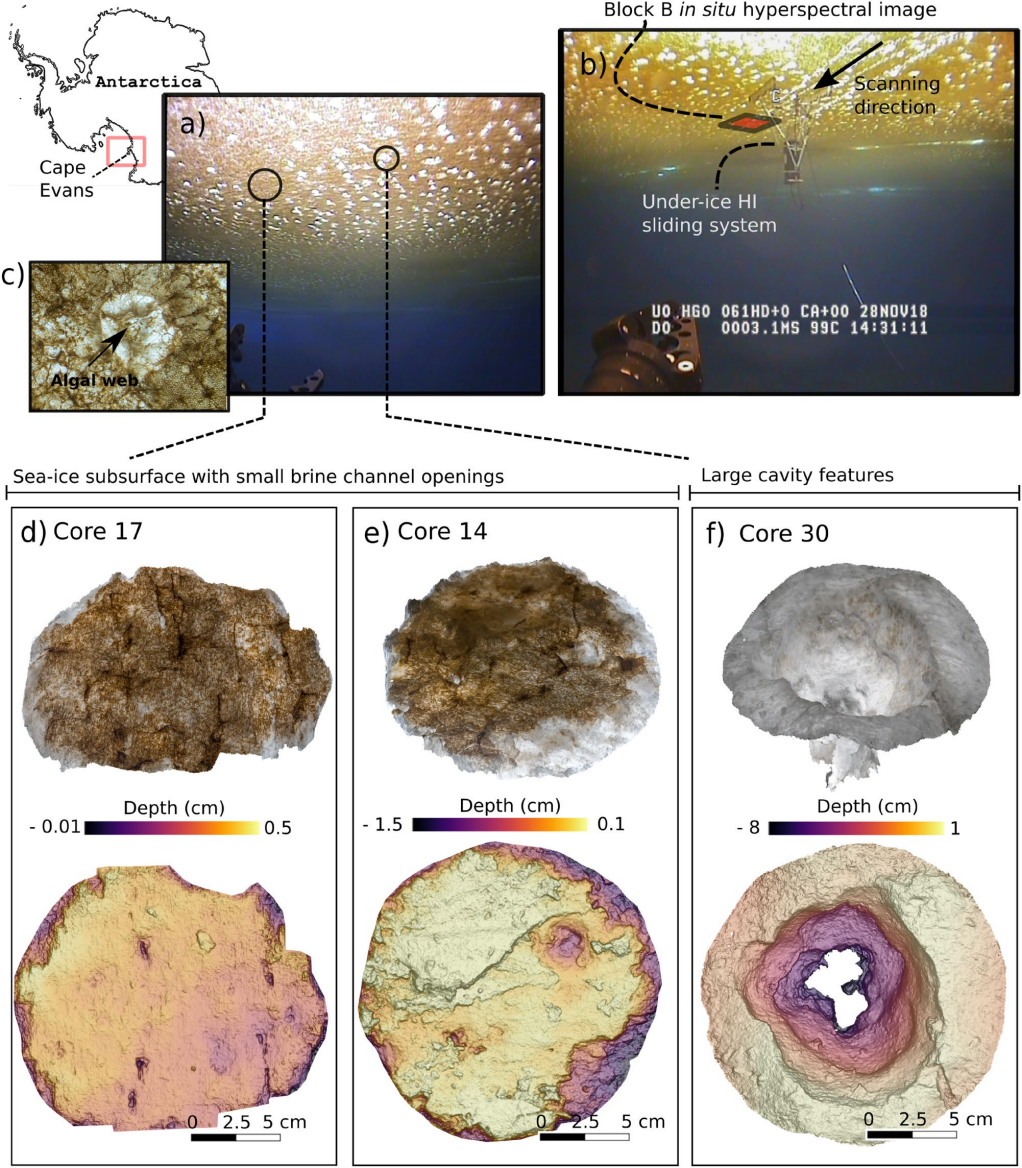
Illustration of a range of biophysical characteristics of the Cape Evans Antarctic fast-ice study site and under-ice hyperspectral image acquisition. (**a**) The distinct under-ice habitat encountered during Spring 2018 was characterized by scattered large cavity features varying widely in diameter and depth. (**b**) Block B under-ice image location and acquisition using the under-ice HI sliding system^38^. (**c**) one of the large cavity features from block B comprising a view of an algal-web like feature stretched on top of large ice cavity. These features on top of the cavity channels were a fairly common feature in the study area. (**d**), (**e**) and (**f**) display an oblique view of the bottom cores surface 3D models (top) and the complex microspatial variability of the under-ice structural features (below). Skeletal layer characteristic of land-fast sea ice is visible along with scale of observable brine channels and cavities.

The under-ice habitat was characterized by a random pattern of large cavities and brine channel openings ranging 3–15 cm in diameter (Fig. 3a). From standard ROV surveys, this pattern was observed up to 200 m from the deployment ice hole in all directions, although we observed sea-ice patches that portrayed little to none of these features (Fig. 3a). Figure 3b illustrates the location of the block B image within the transect of the sliding under-ice HI system^25^. Photogrammetric analyses applied to the bottom of the ice-core surfaces successfully produced scaled 3D models of the under-ice microtopography and revealed unprecedented detail on ice cores microstructure and associated biomass patchiness (Fig. 3d–f). Observed features included the sea-ice skeletal layer (characteristic of growing fast-ice), together with sub-cm sized brine channels (Fig. 3d,e). Figure 3e illustrates a large-scale brine channel of 2.5 cm in diameter together with complex microscale features associated with algal clumps, and other relief features likely associated with localized refreezing events. Figure 3f captures one of the large cavities, which varies from 8 to 10.7 cm in diameter and had a depth of 9 cm. The processes responsible for the formation of these large cavities remain unknown at this stage, as per lack of complementary physical data. However, in addition to these distinct topographical features, the site harboured two different types of algal assemblages making the study site very suitable to showcase the potential of the methodology for capturing fine-scale biomass gradients. The ice algae community was dominated by two diatom species, *Nitzschia stellata*, an interstitial species, and *Berkeleya adeliensis* a strand forming species. *Nitzschia stellata* dominates the interstitial lamellar structure of the ice (Fig. 3d,e) while *Berkeleya adeliensis* forms short strands and aggregates held together by extracellular polymeric substances^56^. Such strands are often visible stretching across the large cavities in web-like formations (Fig. 3c). Strand communities were interspersed among the more diffusely distributed interstitial communities and are associated with dark spots or clumps visible across the oblique images of the ice-core 3D models (Fig. 3d–f).

### PCA for mapping microscale biomass variability in sea-ice cores

**Figure 4:**
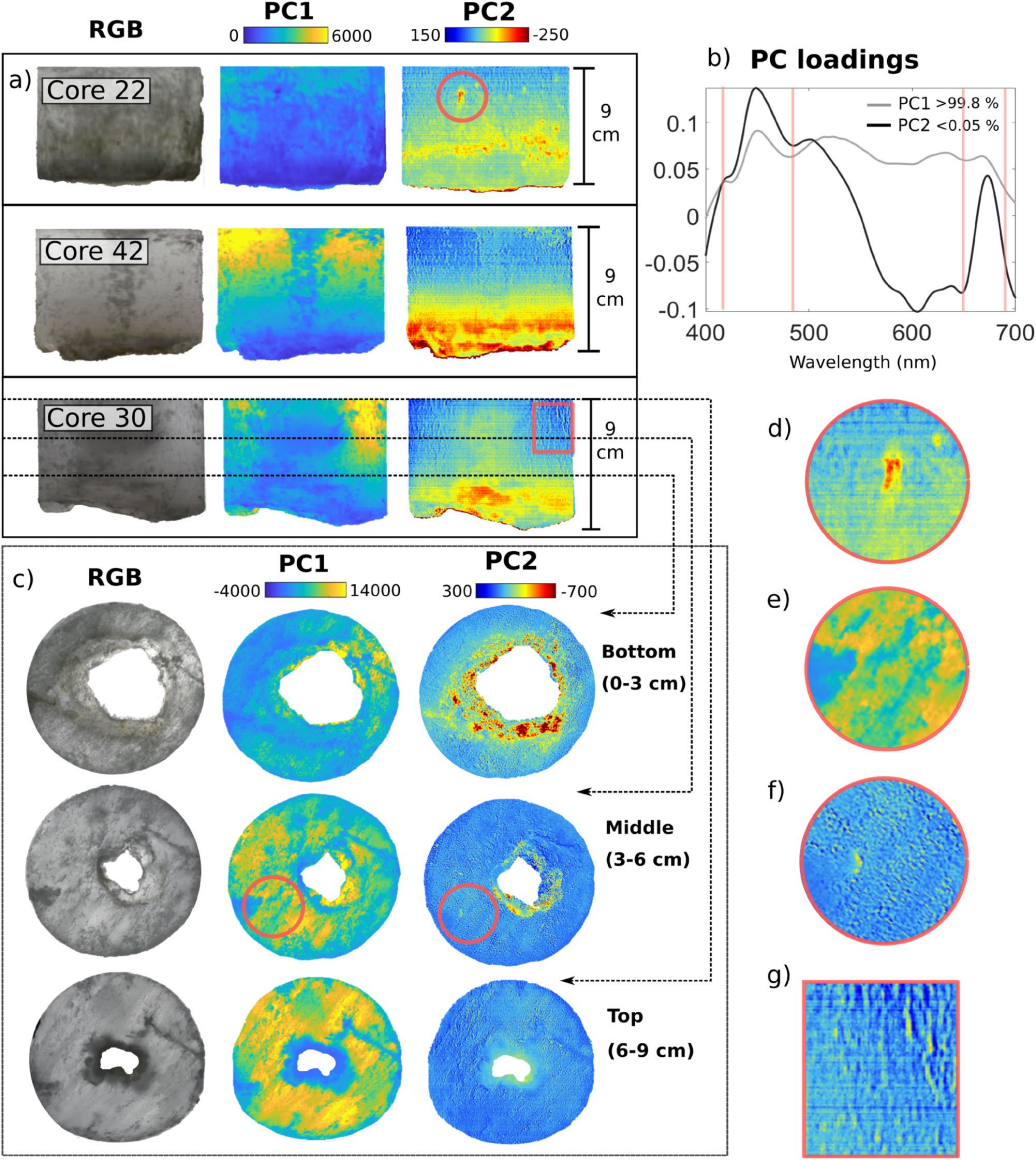
Results of unsupervised exploration using Principal Component Analysis on selected ice core sections. PC analysis was performed independently on pooled vertical and horizontal sections separately. (**a**) three selected vertical scans of cores 22 and 42 are shown alongside core 30, which contained a large cavity that was further explored with horizontal scans of bottom, middle and top sections. (**b**) PCs loadings intensity (unitless). PC1 accounts for > 99.8% of variation and loadings exhibit the spectral signature of the light source thus provides a proxy of transmitted light intensity. PC2 accounts for < 0.05% of variation and loadings are strongly associated with the Chl *a* absorption spectrum. Scores of PC2 map are a proxy of Chl *a* within the cores vertical and horizontal dimensions. (**c**) Horizontal scans and analysis of core 30 characterized by a large cavity feature (see Fig. 3f). The horizontal sections were sliced following the vertical scan. Panels (**d**), (**e**) and (**g**) are zoomed views of selected features of interest such as apparent brine pockets and channels inhabited by algae. (**e**) zoomed view of apparent ice textural properties. RGB composites of the ice core sections were produced using bands at wavelengths 647 nm, 554 nm and 462 nm respectively.

The PC analysis applied to both vertical and horizontal sections of selected ice cores is shown in Fig. 4.

The spectral shapes of the PC loadings derived separately from the vertical and horizontal ice core scans matched almost exactly with differences in loadings of < 0.001% and were averaged for display (Fig. 4b). PC1 accounted for > 99.8% of variability and loadings represented the shape of the solar LED spectrum used for image acquisition (spectrum shown in Fig. 1e). Per-pixel scores of PC1 consequently mapped variability in light intensity transmitted through the core and could therefore be used as a proxy of ice transparency (Fig. 4a,c). PC2 loadings explained < 0.05% of variability (Fig. 4b) and the loading factor closely resembled the Chl *a* absorption spectrum with absorption peaks in the 440 and 670 nm bands^21,57,58^. Due to PCs orthogonality, the influence of variability in light intensity results dampened in PC2 score plot. Thus, PC2 score plots portray a good proxy of Chl *a* concentration over the vertical and horizontal sections of the ice cores, bypassing the need of image normalization (Fig. 4a,c). The impact of the core’s cylindrical geometry, which induces inhomogeneity in light intensity being transmitted across the core width, is also reduced through orthogonality following the first PC rotation. Additional PCs did not display any discernible spectral or spatial patterns of relevance.

PC decomposition is commonly employed in hyperspectral image processing to detect features of interest or for reducing the dimensionality of the data. Here, PCA provided an unsupervised and straight-forward semi-quantitative approach to retrieve and assess proxies of Chl *a* distribution in vertical and horizontal ice core sections without the need of any complementary pigment data (Fig. 4). PCA results from the vertical core scans showed that PC1 proxy of ice-core transparency (Fig. 4b), likely associated with its textural classification and brine volume (e.g., fluid content) but also in part with the amount of biomass. For example, as we approach the very bottom of the core (at the ice water interface) the skeletal layer separates into individual ice lamellae and the ice becomes more porous and coarse. The high permeability of the skeletal layer causes considerable brine loss during the retrieval of the ice core. As the brine leaves the permeable layer, air enters the brine channel system and an increase in scattering occurs, resulting in less light being transmitted. The horizontal PC1 perspective in Fig. 4c,e shows how light transmission seems to decrease (from yellow to blue) as we move down to the bottom of the core (from 9 to 0 cm) or across the core surface, consistent with an increase in lamellar ice texture in the skeletal layer.

The PC2 score maps of the vertical sections showed the fine-scale vertical distribution of ice algal biomass (Fig. 4). As expected, highest densities of Chl *a* were observed in a very thin biofilm at the bottom (ice-water interface) of the skeletal layer (Fig. 4a). Additionally, different microscale patterns can be observed further up into the vertical sections; particularly within the first 0–3 cm of more permeable ice in the skeletal layer. PC2 maps of core 30 (Fig. 4a) displayed a higher biomass within the large cavity relative to the rest of the core. Analysis of the horizontal sections of this core shows a decreasing biomass trend as the cavity narrows (Fig. 4c). This pattern is likely attributed to the surplus habitable ice surface that is exposed to the nutrient rich seawater. Nonetheless, the sampled biomass of core 30 was on the lower end of the biomass scale, compared to the rest of bottom cores samples, which may be a result of localized brine flushing. PC2 loadings of both vertical and horizontal ice core scans further illustrated the widespread occurrence and effects of brine pockets, channel openings and other brine-channel related structures (Fig. 4d,f,g) on ice algal biomass micro-scale distribution. Ice algae thrive within these complex permeable networks^1,2^ and their presence is highly correlated with sea-ice porosity and habitable pore space^59,60^. Although no complementary physical data (temperature, salinity) are available at this scale for validation, PC2 was able to illustrate a limited range of physical features through the ice algal biomass associated with them. We acknowledge that the sea ice analysed in this study was characterized by a relatively simple composition of organic material, mostly microbial derived, and relatively simple translucent and columnar texture common for land-fast ice. Therefore, PC analysis was able to separate the variability in the transmitted light field from the variability due to Chl *a* absorption. Sea-ice textural and structural properties can however vary considerably depending on the growth regime^6^. Consequently, separation through PCA might not be as straightforward in ice cores with diverse sea ice textural mixtures (e.g., granular versus columnar), or with highly variable sea-ice biogeochemical compositions (e.g., high detritus, sediments and coloured dissolved organic matter concentrations).

Unsupervised exploration with PCA opens the potential for investigating complex vertical biophysical patterns and dynamics. For example, understanding how algae migrate through the ice whilst it is being formed and grows^8,61^ and how established bottom ice algae respond to bottom ice growth and ablation. Combining these images with high resolution spatio-temporal temperature and salinity data, will permit for habitable space to be examined alongside nutrient fluxes. This will allow us to better understand how they impact on the vertical variability of algae distribution throughout the sea-ice season^43,60,62^. The methodology could also be applied to explore how ice algae directly respond to changes in their environment through vertical migration following either self-shading or unfavourable light conditions^63^. Capturing such fine-scale patterns using this HI method is a more efficient and quantitatively accurate than cutting ice cores using conventional methods, e.g. sawing. It also permits the extension of the imaging area to larger and more customizable ice sections compared to pulse-amplitude-modulation (PAM) fluorescence imaging techniques^15,64^.

### Evaluation of spectral indices for ice-algal biomass

Previous HI studies in sea ice have focused on assessing HI suitability for ice algal habitat mapping but lacked the availability of quantitative relationships applicable to our particular sensor configuration and study environment^24,25^. The ice core scanning method presented here permits the investigation of bio-optical relationships between traditional and alternative spectral indices (NDIs and continuum removed AUC_650–700_, ANCB_650–700_, ANMB_650–700_ and LAUC_650–700_) against extracted Chl *a* values.

**Figure 5:**
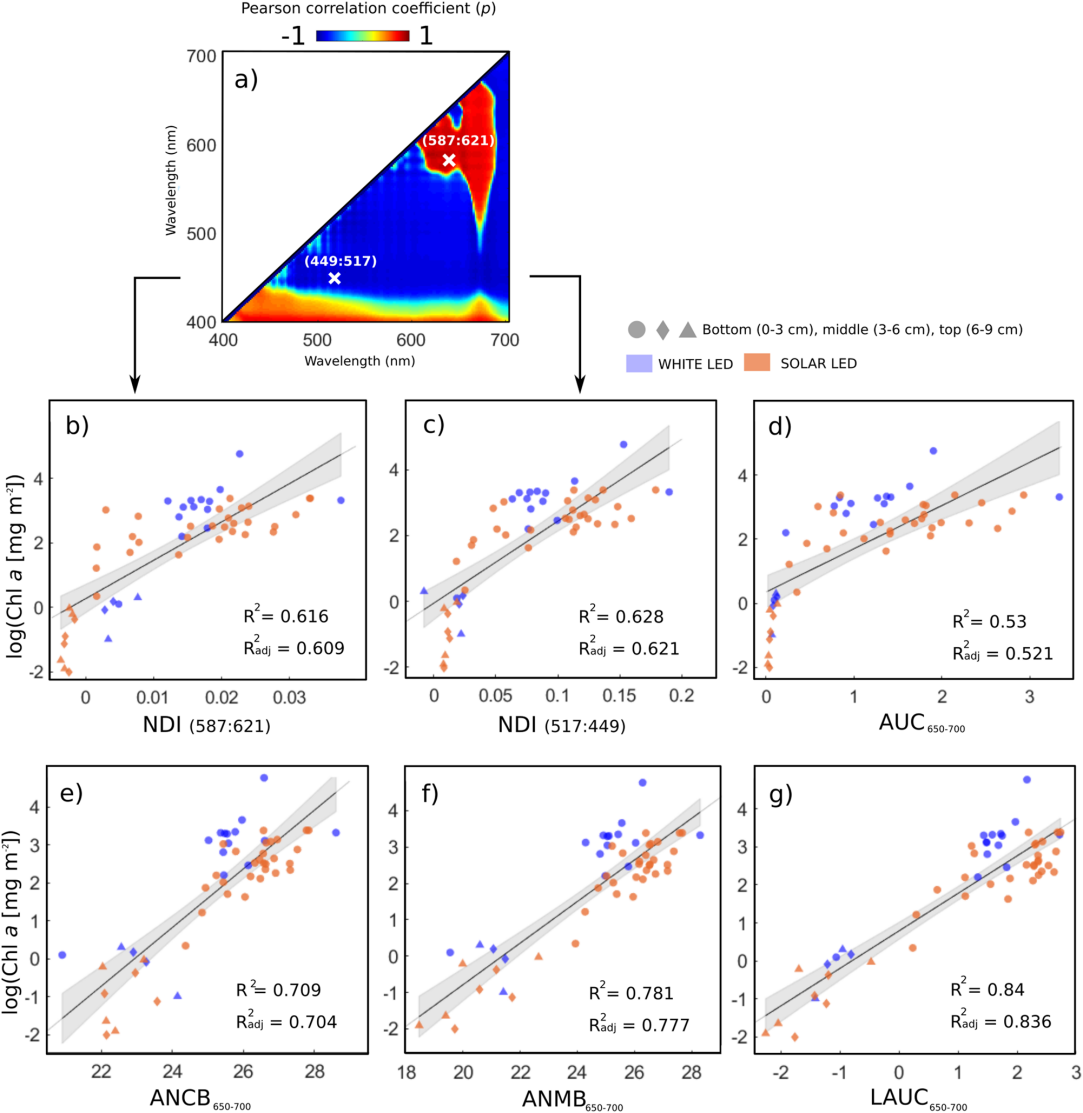
Linear regressions between log-transformed fluorometric chl-a values and derived spectral indices using index computation method (2). Panel (**a**) shows the Pearson correlation surface between all NDIs waveband combinations and Chl *a* values displaying the selected optimal wavelengths. (**b**) and (**c**) illustrate NDI(587:621) and NDI(517:449) tested against sampled Chl *a*. (**d**), (**e**), (**f**) and (**g**) display regression performance of newly developed integrative spectral indices when tested against sampled Chl *a*. The vertical location of the sample (e.g., bottom, middle, top of 9 cm core) and the utilized light source (white or solar LED) is also highlighted in the regression plots to assess any influences on the derived bio-optical regression equations. Regressions lines include 95% confidence interval of the coefficients (shadowed grey areas).

The selected optimal NDI wavelength combinations based on NDI Pearson’s correlation surface and selections criteria were NDI (587:621) and NDI (517:449) (Fig. 5a) and resembled correlation surfaces shown in previous studies^20^. The relationships between tested spectral indices and log(Chl *a* [mg m^−2^]) are shown in Fig. 5b–g together with corresponding regression lines and 95% confidence intervals.

While all indices resulted in significant correlations (R^2^ > 0.5), ANCB_650–700_, ANMB_650–700_ and LAUC_650–700_ indices performed considerably better than both NDIs and AUC_650–700_ for our study case (Fig. 5). Table 1 summarizes the regression and cross-validation details for the tested spectral indices and the derived biomass retrieval parameters. Considering both regression and CV parameters, LAUC_650–700_ outperformed the rest of the indices in retrieving Chl *a* abundance (84% variance explained) from ice-algal assemblages. Despite being able to produce good correlations, both NDIs and AUC_650–700_ seemed to suffer considerably from index saturation at the medium to high biomass values relating mainly to the bottom core sections that dominated the dataset (> 1 log[Chl *a* mg m^−2^]). The ANMB_650–700_ and ANCB_650–700_ indices performed relatively better (Fig. 5e,f and Table 1). We found that the use of spectrally different artificial light sources did not affect the retrieval of coherent correlations following normalization to transmittance (Fig. 5).

**Table 1:** Results of analyses using linear regressions models for estimating Chl a in sea ice based on index computation method (2) (seen in Fig. 2).

Spectral index	α	β	Calibration				Cross-validation (CV)	
			R^2^	RMSE	R^2^_adj_	AIC	MSE_cv_	RMSE_cv_
NDI(587:621)	0.269	119.186	0.629	1.005	0.622	155.771	1.173	1.083
NDI(517:449)	− 0.056	24.927	0.617	1.021	0.610	157.484	1.128	1.062
AUC_650–700_	0.353	1.352	0.530	1.131	0.521	168.465	1.370	1.170
ANCB_650–700_	− 17.718	0.772	0.710	0.889	0.704	142.454	0.871	0.933
ANMB_650–700_	− 12.211	0.571	0.782	0.771	0.777	127.122	0.607	0.779
LAUC_650–700_	0.791	0.986	0.840	0.660	0.837	110.327	0.441	0.664

α and β refer to the regression model intercept and slope found in Eq. 8. R^2^ refers to the coefficient of determination, RMSE stands for Root Mean Square Error, AIC to Akaike Information Criterion.

For our sampled sea-ice cores, the high Chl *a* absorption associated with biomass was more pronounced around the 650–700 nm part of the spectrum, compared to 440–450 nm, where noise was dominant (Fig. 2b). This is attributed to the highly concentrated bottom algal layer that along with a 1.8 m thick ice cover, reduced light levels considerably to $E_{{d,400 - 700\,{\text{nm}}}}$ = 0.35 ± 0.20 W m^-2^, particularly in the 400–500 nm visible range of the spectrum (Fig. 2b). We speculate that the performance of the ANCB_650–700_ and ANMB_650–700_ benefitted from the lack of snow cover. Snow is a strong absorber above 600 nm^51,65^ and its presence is expected to have a negative influence on the retrieval of relationships for the 650–700 range of the spectrum. A potential limitation of the ANCB and ANMB indices could be the inability to retrieve low chlorophyll abundance values as the spectral influence of background features predominates^49^. Log-transformation of the integrative index AUC_650–700_ into LAUC_650–700_ provided the best retrieval for Chl *a* abundance, because it inherently accounts for the exponential decrease in light passing through an absorbing medium. This consideration, as also seen for sedimentary algal habitats^33^, along with the log-transformation of the extracted Chl *a* concentrations produces the most evenly distributed linear spread of spectral index and Chl *a* values (Fig. 5g).

In recent years, bio-optical algorithms capable of mapping biomass under-ice have been derived from cosine corrected irradiance sensors deployed via L-arms followed by the extraction of overlapping core samples to produce series of regression points^10,48^. However, existing relationships retrieved from sensors which integrate radiance over large solid angles and greatly differ in SNR, are arguably not compatible with the per-pixel radiance signals from fine scale HI pixels^16^. In addition, NDIs have not always been able to produce robust correlations, or derived relationships are limited in their transferability between study sites and between seasons^16,21,22,48^ . This is because differences in sea-ice and snow physical properties and ice algae photophysiological conditions (e.g., pigments composition and packing), can change the optical pathway of light considerably^9^. This has an impact on the retrieved model coefficients thus affecting the robustness of model to be applied onto new datasets^22,48^. For nearby sites, recent studies have struggled to formulate reliable bio-optical regression models^16,21^. This was attributed mostly to the presence of platelet ice, which results in considerable biomass losses during sampling and consequently narrow biomass variability range. Another reason could be attributed to the particularly high biomass concentrations found in the fast-ice of McMurdo Sound^2^, because high biomass concentrations can negatively affect linear relationships through the saturation of various vegetation spectral indices^49,66,67^.

Through the development of an ice-core HI approach and alternative spectral indices, we were able to compensate for some of the caveats relating to sample collection and model development. This core scanning method also allowed the elaboration of relationships that are more suitable to be applied to imagery from high-resolution HI sensors. Results cannot be compared with previous studies in the same area using L-arms, as sea-ice conditions were drastically different^16,21^ and the light-sample collection procedures were also noticeably different. HI on extracted ice cores poses a distinct advantage in that the spectral data specific for Chl *a* is extracted in full. The differences between the in situ spectral measurements and the standard biological processing that are caused through to the process of coring, brine drainage or platelet slough-off result therefore minimized. Another advantage of the spatial resolution of the system is that we can precisely contour relevant per-pixel radiance and operate within the exact surface area that is being sampled for Chl *a* extraction.

To improve ice algal biomass estimation models, it will remain critical to be able to sample Chl *a* at scales closer to the optical resolution of HI scanning systems so that small features can be referenced in the HI products. In our case, the variance in Chl *a* samples was reduced as we averaged over the entire ice core surface of 14 cm-diameter which portrayed high biomass gradients within single ice core surfaces (Fig. 3). We could partially account for the range reduction by including the horizontal core sections sectioned beyond the lower-most 3 cm section (e.g., 3–6, and 6–9 cm sections). This permitted the quantification of ice algal biomass over a wider range of concentrations also found in situ (e.g., areas surrounding large cavities and bare ice spots) (Fig. 3). However, bottom Chl *a* concentrations can range widely in sea ice, with integrated values reported for Antarctic fast-ice ranging between < 0.1 up to 219 mg m^−2^^4^, thus further efforts are needed to enhance baselining techniques.

As ice science continues to create ever-growing datasets, integrating samples from multiple seasons and areas will help to increase the robustness of the algorithms through statistical learning models taking advantage of the potential of the resolution of hyperspectral systems.

### Quantitative mapping of Chl a microspatial variability ex situ and in situ

**Figure 6:**
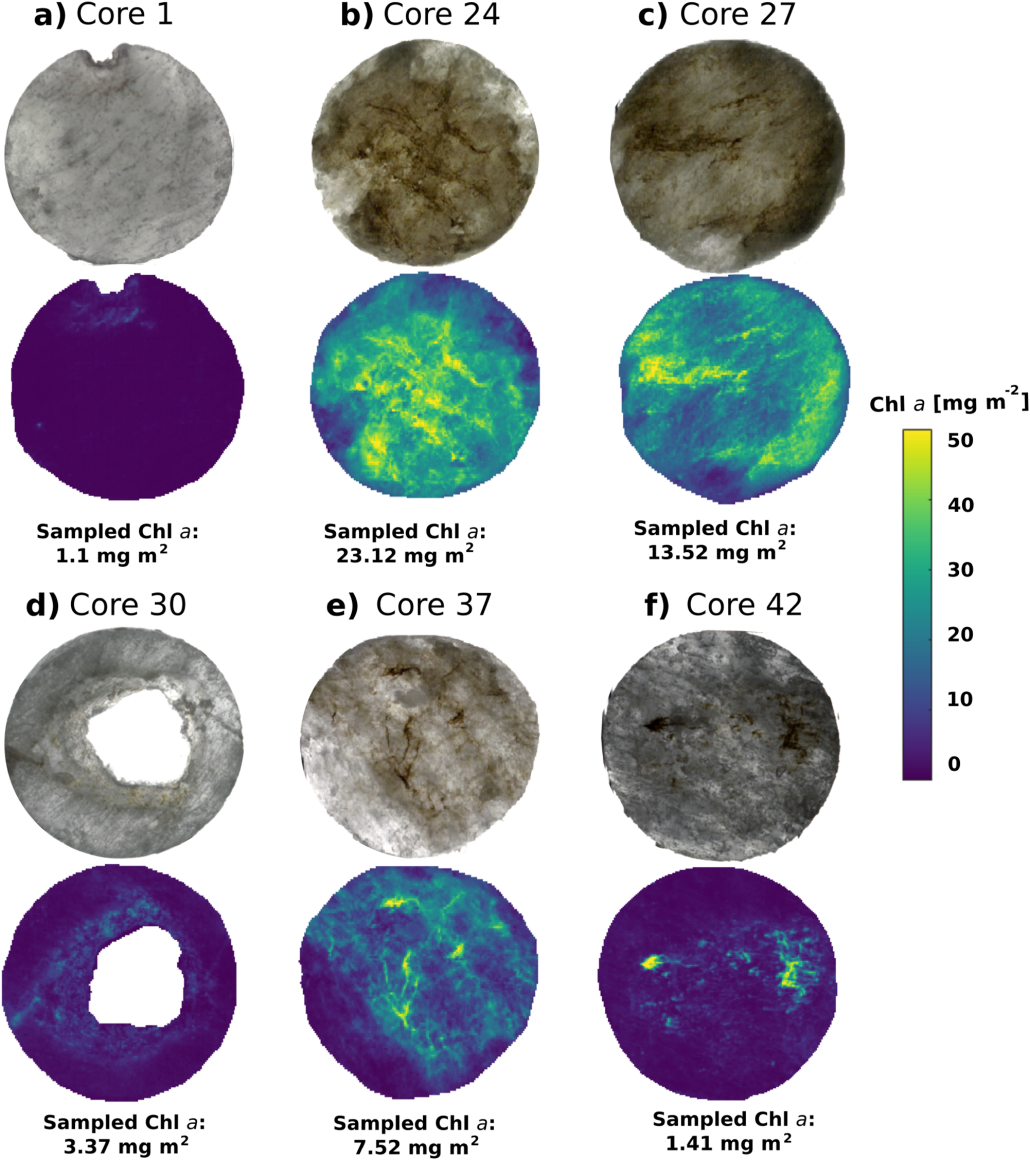
Application of best performing bio-optical regression model to the ex situ imagery of selected horizontal sea-ice core sections (below) along with their respective RGB composite (top). Best performing linear model was derived using the LAUC index (or log(AUC_650–700_)) (see Table 1). Fluorometrically derived concentrations of extracted Chl *a* value of each core section are provided to compare extracted Chl *a* with the optically quantified Chl *a* values. High variability in biomass abundance can be observed within the 0.015 m^2^ (ø 14 cm) core surfaces and across different cores in a spatially explicit manner*.* RGB composites of the ice core sections were produced using bands at wavelengths 647 nm, 554 nm and 462 nm respectively.

**Figure 7:**
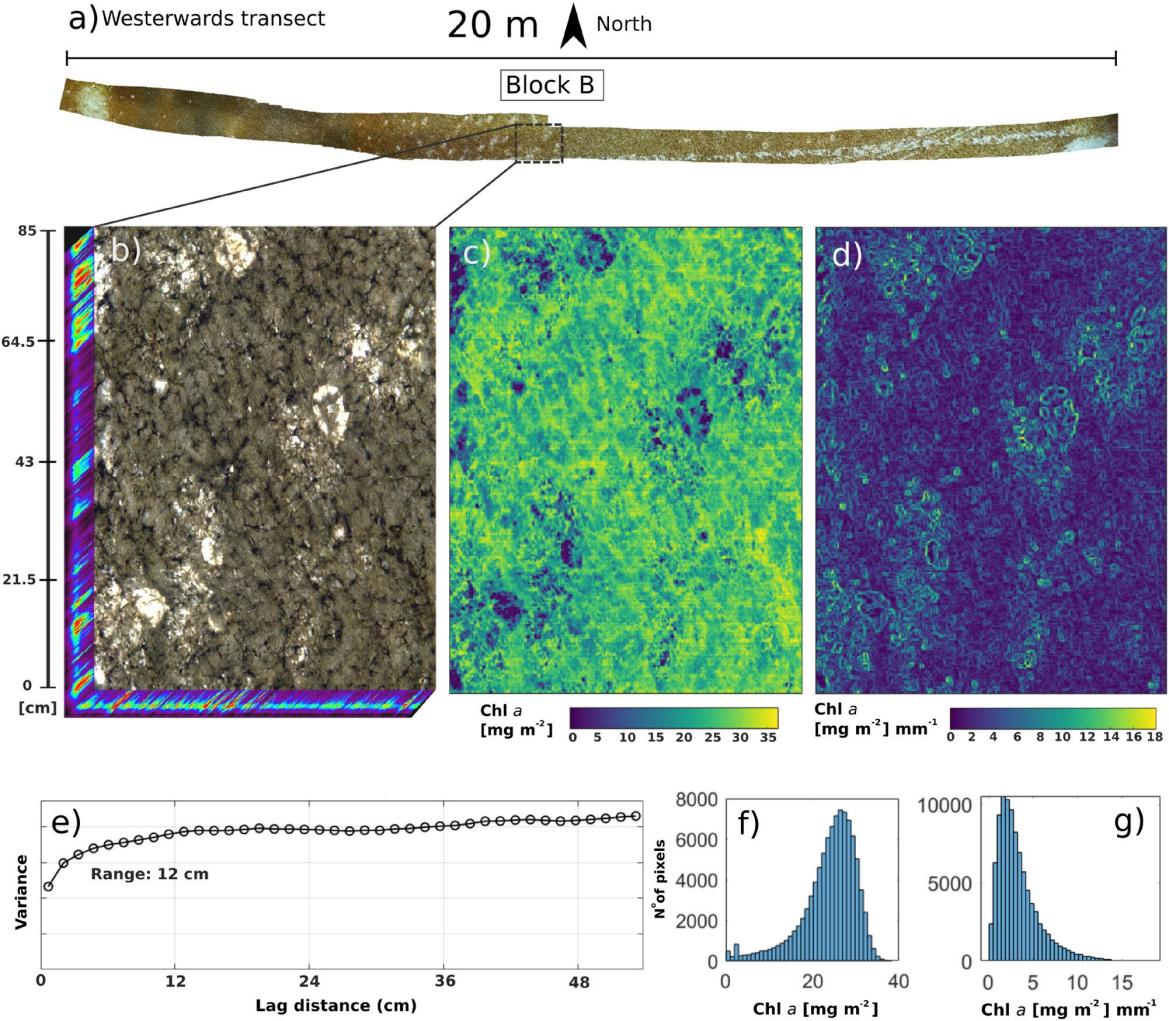
Application of best performing bio-optical regression model (LAUC) to the in situ under-ice imagery and associated microspatial analyses. (**a**) Framing of the block B hyperspectral image subsample within the entire 20 × 0.6 m transect provides an indication of the spatial scale. (**b**) A high-resolution HI data cube representation over block B which is 0.85 × 0.7 m in extent. (**c**) A quantitative mapping of Chl *a* by applying the LAUC index regression model on a per pixel basis to the pseudo-transmittance image of block B. Spatial resolution following a × 4 binning is 2.4 mm per pixel. (**d**) Magnitude of gradient of Chl *a* concentration derived from block B using a Prewitt kernel. (**e**) Empirical variogram of ice algal biomass computed by randomly sampling 10,000 pixels within the image and starting with a lag distance of 1.2 cm (5 pixels). Variances are shown in arbitrary units starting at zero. (**f**) Histogram of per-pixel Chl *a* estimates over entire block B shown in panel **c**). (**g**) Histogram of per pixel gradient distributions relative to gradient map shown in panel **d**). RGB composites of the under-ice hyperspectral imagery was produced using bands at wavelengths 647 nm, 554 nm and 462 nm respectively.

The optimal predictive linear model built on LAUC_650–700_ was applied on a per-pixel basis to both ex situ imagery from the retrieved horizontal ice cores sections (Fig. 6), and to in situ imagery of block B retrieved with our under-ice scanning system (Fig. 7). The resulting quantitative maps of Chl *a* (mg m^−2^) characterize the remarkable patchiness of sea-ice algal biomass at the micro and macro scale (Figs. 6a–f and 7c). Previous surveys of biomass variability have been quite discrete in sampling resolution (e.g., 0.5–2 m eters distance at the finest)^18,22^. Apart from ice coring surveys, broader footprints derived from different cosine corrected sensor types necessarily integrate in signal variance and therefore in biomass variability.

For example, under-ice trawl-based approaches allow to capture kilometre scale transects at the cost of resolution, with footprints averaging tenths of meters in length^68^. Currently no means have been developed to apply HI at the mesoscale. However, the HI niche is its capability to capture and parametrize variability in sea-ice biophysical properties at unprecedently fine scales, which will help to understand microspatial scale processes characteristic of sympagic microalgal community dynamics^15,62^.

Spatial variability within single ice-core surfaces showed highly heterogeneous patterns (Fig. 6a–f), even when compared to the < 1 m^2^ image of block B (Fig. 7c). At the same time, Chl *a* variability measured across cores taken less than 10 m apart varied by one order of magnitude as seen in Fig. 6b and d between the large cavity core 30 (3.37 mg m^−2^) and core 24 (23.12 mg m^−2^). Variogram analyses revealed a Chl *a* autocorrelation length scale of about 12 cm (see range in Fig. 7e). This was related to habitat features of this size such as the network of algal clusters and the cavities (observed in Fig. 7b,c). However, the variogram also underlines a relatively high nugget to sill effect, suggesting the influence of yet smaller scale variations in Chl *a* estimates. These were attributed to a combination of measurement error (pixel noise) and stark gradients occurring at the mm scale. The gradient intensity map shown in Fig. 7d highlighted drastic Chl *a* abundance gradients, with a mean of 3.3 ± 2.3 mg m^−2^ mm^−1^ and reaching up to a maximum of 18 mg m^−2^ mm^−1^ (see histogram in Fig. 7g).

Regarding validation, the average pixel-based biomass estimate for each of the six core samples was consistent with its respective sampled value, with a variability of about 25–35%. The predicted Chl *a* concentration on block B shows estimates consistent with the range of sampled values for our study site (Fig. 7f and Supplementary Fig. 1). However, it remains challenging to validate high-resolution under-ice HI products (2.4 mm size pixels) as comparative methodologies to collect physical samples at the scales are lacking. Common ice coring devices can only sample up to a certain diameter size and biomass losses associated with coring (particularly of loose strand-forming diatoms) will have to be accounted for when validating estimates. PAM fluorescence imaging could offer some ground truthing although it can only provide biomass proxies for considerably smaller frames sizes (from 30 × 23 mm up to 120 × 70 mm) and currently exclusively on extracted cores only^64^. Therefore, traditional sampling of Chl *a* over smaller surface areas that are referenceable within the HI products should be pursued to support the validation aspect of the method.

We note that the applied relationships are derived by scanning the 3 cm thick layers only, and the remaining ~ 177 cm of the ice core were omitted from our analyses. This is considered a suitable approach for our study area as > 98% of the biomass was concentrated within the bottom 3 cm of the ice ; a common feature for the fast ice off Cape Evans^4,69,70^. In situ imagery was converted to pseudo-transmittance images through normalization by the radiance coming from the cavity and bare ice features within the scene (Figs. 2 and 7b). There are however different sea-ice types with more pronounced variations in vertical biomass variability^2,8^ and also sea ice that does not exhibit cavity features that can be used for estimating in situ transmittance. The workflow presented here could therefore require modification for different ice environments such as the integration of scans from core sections higher into the ice core, artificially coring cavity-like features, or normalizing irradiances using simple radiative transfer models^16,71,72^.

Although still under development, we underline that HI approaches present major opportunities for microspatial mapping of ice algal biomass in situ, and ex situ across different vertical and horizontal ice core sections. We further advocate the potential for the methodology, coupled with RGB imaging, to be mounted onto UUVs or stationary monitoring stations to drastically increase the spatial and temporal mapping capability of under-ice biophysical dynamics quantitatively^28,34,73^. The selected study site in particular showcased the need for adequate resolutions whereby biophysical properties of the under-ice habitat (e.g., changes in species composition and under-ice topography) were shown to lead the spatial biocomplexity portraying stark small-scale biomass gradients (Fig. 7d). For example, the observed cavities can provide additional surface area or shelter for the algae to colonize, however encompass radiance levels that are orders of magnitude higher than the average conditions, with unknown effects on surrounding microorganisms. When combined with photogrammetric 3D models, fine scale biomass mapping can support an improved understanding of the effect of under-ice topography, lamellar ice crystal orientation and roughness on sea ice algal biomass patchiness^15,25,59,74^. At the boundary layer, the effects of shear stress from underlying currents and nutrient exchange processes on microscale biomass variability remain understudied and could be addressed with HI. Fine scale sea-ice biophysical dynamics can further complicate the causation effects if we consider that ice algal growth can potentially create a feedback to changes in sea-ice physical properties through heat absorption and melting^75^ or through extracellular polymeric substances (EPS) production affecting the sea-ice microstructure^76^. Grazing from pelagic herbivores can also influence patchiness and distribution although little is known about their quantitative influence at any scale. Time-lapse approaches coupling under-ice HI and RGB systems^25^, integrated with ice core scanning, could further help assessing grazer-biomass interactions at relevant spatial scales. In addition, through concomitant monitoring of both sea-ice microstructural properties, biomass dynamics and their environmental drivers over time, the method could further support and add to the parameterization of sea-ice biogeochemical and ecological models aimed at regional scale analyses.

## Supplementary Information

Supplementary Information.
