# Supplementary material for: Mapping the in situ microspatial distribution of ice algal biomass through hyperspectral imaging of sea-ice cores
Source: Sci Rep. 2020 Dec 14;10:21848. doi: 10.1038/s41598-020-79084-6 (PMC7736878; doi:10.1038/s41598-020-79084-6)
Supplement: Supplementary file 1 — Supplementary Information. [file 41598_2020_79084_MOESM1_ESM.pdf]

Supplementary material

Mapping the *in situ* microspatial distribution of ice algal biomass through hyperspectral imaging of sea-ice cores

Emiliano Cimoli [1], Vanessa Lucieer [1], Klaus M. Meiners [2,3], Arjun Chennu [4,5], Katerina Castrisios [1], Ken G. Ryan [6], Lars Chresten Lund-Hansen [7,8], Andrew Martin [1], Fraser Kennedy [1], Arko Lucieer [9]

[1] Institute for Marine and Antarctic Studies, College of Sciences and Engineering, University of Tasmania, Private Bag 129, Hobart, Tasmania 7001, Hobart, Australia
[2] Australian Antarctic Division, Department of Agriculture, Water and the Environment, Kingston, Tasmania 7050, Australia
[3] Australian Antarctic Program Partnership, Institute for Marine and Antarctic Studies, University of Tasmania, Hobart, Tasmania 7001, Australia
[4] Max Planck Institute for Marine Microbiology, Celsiusstr. 1, Bremen 28359, Germany
[5] Leibinz Center for Marine Tropical Research, Fahrenheitstrasse 6, Bremen 28359, Germany
[6] School of Biological Sciences, Victoria University of Wellington, PO Box 600, Wellington, New Zealand
[7] Aquatic Biology, Department of Bioscience, Aarhus University, Ole Worms Alle´ 1, Building 1134, DK-8000 Aarhus C, Denmark
[8] Arctic Research Centre, Aarhus University, Ny Munkegade 116, Building 1540, DK-8000 Aarhus C, Denmark
[9] Discipline of Geography and Spatial Sciences, School of Technology, Environments and Design, College of Sciences and Engineering, University of Tasmania, Private Bag 76, Hobart, Tasmania 7001, Australia

Specification and technical information on the ice core scanning system

The scanning HI set-up consisted of a push-broom hyperspectral imager (Aisa Kestrel 10, Specim Ltd, Finland) mounted onto a RAM 40" motorized rail (Revolve Camera, USA). The optics of the camera (F/2.8, 40° wide) were focused on the top surface of a sample holder beneath the scanner (Figure 1c in manuscript). The imager moves at a constant speed whilst recording the spectral radiance field (from 400-1000 nm at 1.7 nm spectral resolution for a total of 356 bands) across the surface of the ice core sample (approximately 0.45 mm spatial resolution) in a relatively short duration (< 2 minutes according to integration times and rail speed). The imager sensitivity and scanning speed were tuned to maximize transmitted signal and to produce imagery at a coherent geometry and with square pixels. This was achieved by tuning integration time and imaging frequency along with rail speed depending on the ice-core thickness and transparence.

Two different light systems were utilized as the artificial light sources to illuminate the cores. These included a white LED (light-emitting diode), constituting the typical dual peaked spectrum shape (Figure 1d in manuscript) and a solar LED, designed to resemble the solar spectrum (Figure 1e in manuscript). The use of two different LEDs gave us the ability to test the robustness of the method independent of the light source. From these results we can independently assess how scans using different light sources might be combined or compared. The LEDs were set to emit an $E_{d,\,PAR}$ of < 30 µmol photons m^{-2} s^{-1} to avoid potentially photo-damaging the algal communities, which are typically low-light adapted. Reducing the light intensity comes at the expense of the signal to noise ratio (SNR). It does provide us with comparable SNR to *in situ* under-ice imaging environments which were acquired using natural sunlight transmitted through the sea ice.

The illumination provided a diffuse and homogenous coverage of the entire sample. A homogenous light field was achieved by selecting an appropriate distance (20 cm) between the ice core and the light source and placing a semi-opaque white glass substrate diffuser in between (Figure 1c in manuscript). The core sections were placed on top of a 15 x 15 cm acrylic glass tray inside a black box to reduce external light contamination (Figure 1c in manuscript). It is noted that while on our application only the bottom 9 cm of the entire core was scanned (Figure 1b in manuscript), the hyperspectral scanning concept is applicable for any desired core length or section morphologies (e.g., slabs) through adequate modifications of the set-up.

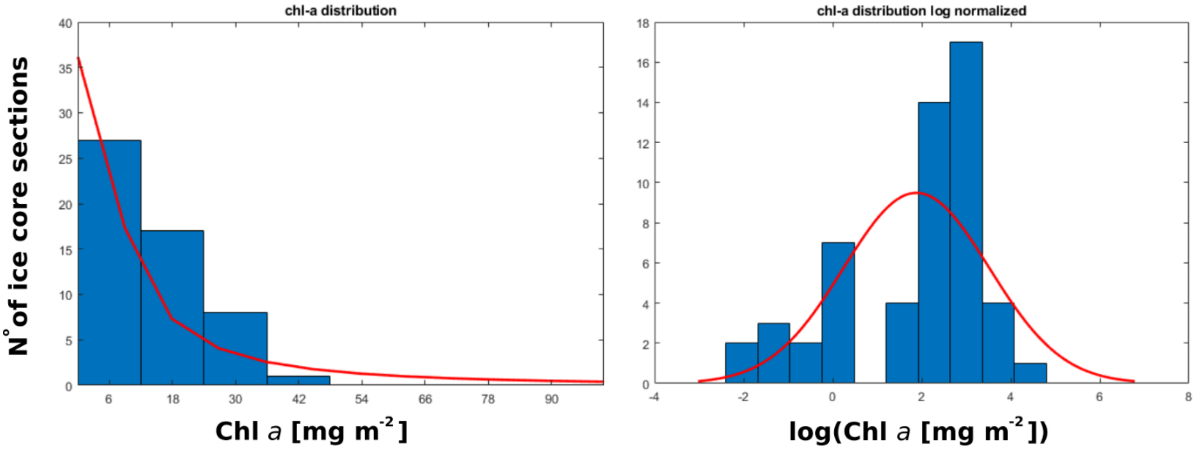

Supplementary Figure 1. Distribution of sampled chlorophyll a (Chl *a*) of all the horizontal ice cores sections (54 samples in total) retrieved during the 2018 Cape Evans Antarctic field campaign. Cores for this experiment were extracted following standard procedures and cut into 3 cm thick sections from 0 to 3 cm (42), and occasionally followed by 3 to 6 cm (6 samples) and 6 to 9 cm (6 samples) as described in the methods sections. Red lines indicate the theoretical distribution function representing the sampled values.

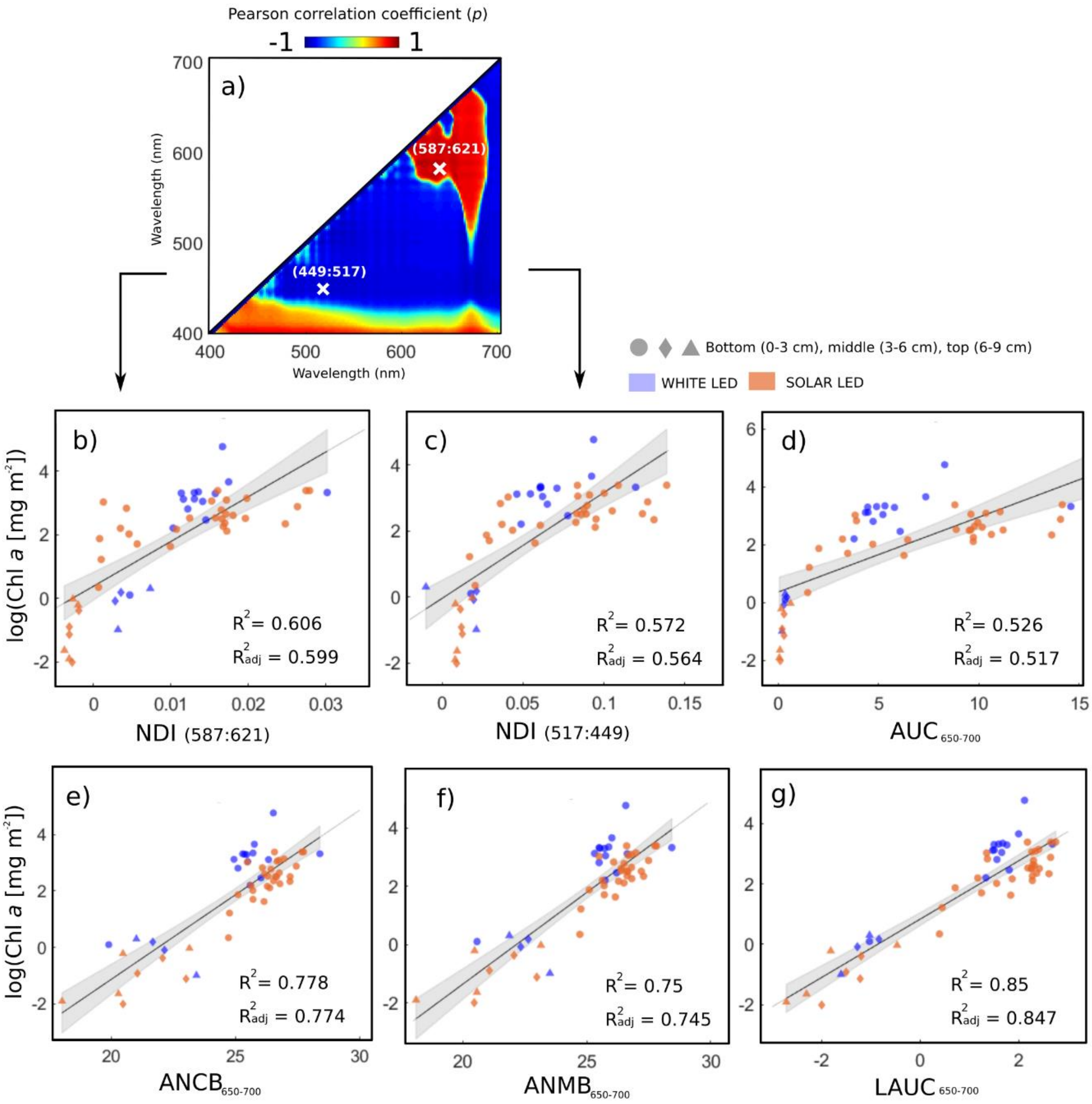

Supplementary Figure 2. In order to retrieve an index value for each core section, two approaches are available (see Figure 2 in manuscript): calculate the spectral index (1) from the mean transmittance spectrum of all pixels of the scanned core or (2) from each of the pixels spectrums in the preprocessed pixel of the core and then calculate the mean. These will not be identical as some of the spectral index formulae are not algebraically commutative. The latter approach involves calculation from multiple noisy spectra (from each pixel) but has the advantage that a satisfactory regression model can then be applied to in situ pseudo-transmittance images and thus was selected in the main manuscript. Linear regressions between log-transformed fluorometric Chl *a* values and derived spectral indices using index computation method (1) are shown in this figure. Panel **(a)** shows the Pearson correlation surface between all NDIs waveband combinations and chl-a values displaying the selected optimal wavelengths. **(b)** and **(c)** illustrate NDI(587:621) and NDI(517:449) tested against sampled chl-a. **(d)**, **(e)**, **(f)** and **(g)** display regression performance of newly developed integrative spectral indices when tested against sampled Chl *a*. The vertical location of the sample (e.g., bottom, middle, top of 9 cm core) and the utilized light source (white or solar LED) is also highlighted in the regression plots to assess any influences on the derived bio-optical regression equations. Regressions lines include 95% confidence interval of the coefficients (shadowed grey areas).

Supplementary Table 1. Results of analyses by the linear regressions models for estimating Chl *a* in sea ice based on index computation method (1) (see previous Supplementary Figure 2 and Figure 2 in main manuscript). We found very little difference in performance between method (1) and method (2). α and β refer to the regression model intercept and slope respectively found in Equation 8 of manuscript. R^2 refers to the coefficient of determination, RMSE stands for Root Mean Square Error, AIC to Akaike Information Criterion.

**Spectral index**	**α**	**β**	**Calibration**				**Cross-validation (CV)**	
			**R^2**	**RMSE**	**R^2_{adj}**	**AIC**	**MSE_{cv}**	**$RMSE_{cv}$**
**NDI(587:621)**	0.376	140.805	0.607	1.034	0.599	158.870	1.123	1.060
**NDI(517:449)**	-0.047	32.094	0.573	1.078	0.565	163.343	1.200	1.096
**$AUC_{650-700}$**	0.361	0.259	0.526	1.135	0.517	168.929	1.376	1.173
**$ANCB_{650-700}$**	-13.952	0.629	0.779	0.776	0.775	127.798	0.622	0.789
**$ANMB_{650-700}$**	-13.128	0.600	0.750	0.825	0.745	134.419	0.703	0.838
**$LAUC_{650-700}$**	0.836	0.966	0.850	0.638	0.848	106.688	0.415	0.644
